# Renoprotective effects of a factor Xa inhibitor: fusion of basic research and a database analysis

**DOI:** 10.1038/s41598-018-29008-2

**Published:** 2018-07-18

**Authors:** Yuya Horinouchi, Yasumasa Ikeda, Keijo Fukushima, Masaki Imanishi, Hirofumi Hamano, Yuki Izawa-Ishizawa, Yoshito Zamami, Kenshi Takechi, Licht Miyamoto, Hiromichi Fujino, Keisuke Ishizawa, Koichiro Tsuchiya, Toshiaki Tamaki

**Affiliations:** 1 Department of Pharmacology, Institute of Biomedical Sciences, Tokushima University Graduate School, Tokushima, Japan; 2 Department of Pharmacology for Life Sciences, Institute of Biomedical Sciences, Tokushima University Graduate School, Tokushima, Japan; 3 Department of Pharmacy, Tokushima University Hospital, Tokushima, Japan; 4 Department of Clinical Pharmacology and Therapeutics, Institute of Biomedical Sciences, Tokushima University Graduate School, Tokushima, Japan; 5 Clinical Trial Center for Developmental Therapeutics, Tokushima University Hospital, Tokushima, Japan; 6 Department of Medical Pharmacology, Institute of Biomedical Sciences, Tokushima University Graduate School, Tokushima, Japan

## Abstract

Renal tubulointerstitial injury, an inflammation-associated condition, is a major cause of chronic kidney disease (CKD). Levels of activated factor X (FXa), a blood coagulation factor, are increased in various inflammatory diseases. Therefore, we investigated the protective effects of an FXa inhibitor against renal tubulointerstitial injury using unilateral ureteral obstruction (UUO) mice (a renal tubulointerstitial fibrosis model) and the Food and Drug Administration Adverse Events Reporting System (FAERS) database. The renal expression levels of FX and the FXa receptors protease-activated receptor (PAR)-1 and PAR-2 were significantly higher in UUO mice than in sham-operated mice. UUO-induced tubulointerstitial fibrosis and extracellular matrix expression were suppressed in UUO mice treated with the FXa inhibitor edoxaban. Additionally, edoxaban attenuated UUO-induced macrophage infiltration and inflammatory molecule upregulation. In an analysis of the FAERS database, there were significantly fewer reports of tubulointerstitial nephritis for patients treated with FXa inhibitors than for patients not treated with inhibitors. These results suggest that FXa inhibitors exert protective effects against CKD by inhibiting tubulointerstitial fibrosis.

## Introduction

There has been a global increase in the incidence of chronic kidney disease (CKD), with a high morbidity and mortality in the general population^1,2^; therefore, it is imperative to elucidate the underlying mechanism and discover novel therapeutic strategies. Renal tubulointerstitial injury, which is associated with increased inflammation, is a major cause of CKD progression^3^.

The progression of CKD is associated with the increased activation of blood coagulation factors^4,5^. Increasing evidence suggests that, acting via protease-activated receptors (PARs), activated factor X (FXa) plays an important role not only in the coagulation cascade, but also in various inflammatory diseases, such as fibrosis^6,7^, atherosclerosis^8,9^, and cancer^10^. Four PARs (1–4) have been cloned and identified to date^11^. FXa can activate PAR-1 and PAR-2, which are expressed in various cell types, such as fibroblasts and endothelial cells^12^. *In vitro* studies have revealed that FXa augments inflammatory molecules, such as monocyte chemotactic protein (MCP)-1, interleukin (IL)-1β, and tumour necrosis factor (TNF)-α in many cell types, including macrophages, endothelial cells, and podocytes^8,13,14^. Thus, targeting FXa can have great clinical significance in terms of the treatment of various inflammatory diseases, including CKD.

FXa inhibitors, also called direct oral anticoagulants, are widely used as alternatives to warfarin for the prevention of stroke in patients with non-valvular atrial fibrillation and for the prevention and treatment of venous thromboembolism. However, the effect of FXa inhibitors on renal tubulointerstitial injury remains unclear.

In this study, we investigated the effect of an FXa inhibitor edoxaban on renal tubulointerstitial injury using mice with unilateral ureteral obstruction (UUO), a well-established experimental animal model of tubulointerstitial fibrosis. To determine the renoprotective effects of FXa inhibitors in humans, we also evaluated data from the Food and Drug Administration (FDA) Adverse Events Reporting System (FAERS) database. This database is one of the largest global databases, containing millions of case reports on drug-associated adverse events, and is routinely used for pharmacovigilance^15–18^ because it provides real-world clinical data.

## Results

### Renal expression of coagulation factors and PARs

**Figure 1:**
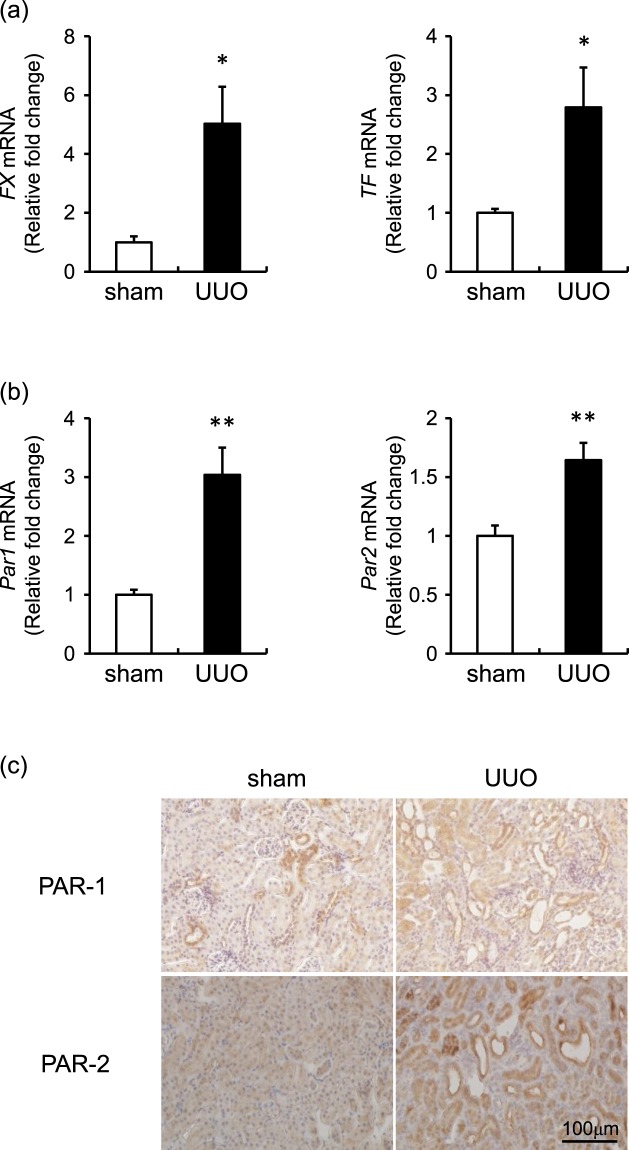
Expression of coagulation factors and protease-activated receptors (PARs) in the kidney. Changes in the mRNA levels of (**a**) *FX* and *TF*, and (**b**) *Par1* and *Par2* at day 7 in the kidneys of mice that received a sham operation with vehicle (sham; white bar) or unilateral ureteral obstruction (UUO) with vehicle (UUO; black bar). The values are expressed as means ± standard error of the mean (SEM). *p < 0.05, **p < 0.01 vs. sham mice; n = 7–8. (**c**) Representative immunohistochemical results for PAR antibodies 7 days after operation.

We first performed quantitative reverse transcription polymerase chain reaction (qRT-PCR) to examine the renal expression of coagulation factors (*FX* and tissue factor [*TF*]) and PARs (*Par1* and *Par2*). Seven days after the operation, the *FX* and *TF* mRNA levels in UUO with vehicle mice (UUO mice) were higher than those in sham operation with vehicle mice (sham mice) (Fig. 1a). *Par1* and *Par2* mRNA levels in UUO mice were also higher than those in sham mice (Fig. 1b). Based on immunochemical staining, PAR-1 and PAR-2 were located mainly in tubules and were expressed more highly in UUO mice than in sham mice (Fig. 1c).

### Plasma FXa activity

Next, we measured plasma FXa activity using enzyme-linked immunosorbent assays (ELISAs). There was no difference in plasma FXa activity between sham mice and UUO mice. Plasma FXa activity was significantly suppressed in UUO with edoxaban mice (UUO + EDO mice) compared with sham mice and UUO mice (Table 1).

**Table 1:** Plasma FXa activity.

	sham	UUO	UUO + EDO
Plasma FXa (ng/mL)	5.6 ± 0.5	5.4 ± 0.9	1.4 ± 0.3**^,##^

Values are expressed as means ± standard error of the mean (SEM). n = 9–14 per group. **p < 0.01 vs. sham; ^##^*p* < 0.01 vs. UUO.

### Effect of the FXa inhibitor on renal function parameters

In UUO mice, creatinine and blood urea nitrogen (BUN) levels are elevated^19^. Therefore, we measured plasma creatinine and BUN to confirm the renoprotective effect of the FXa inhibitor. Plasma creatinine and BUN were significantly higher in UUO mice than in sham mice. The increase in plasma creatinine in UUO mice was significantly inhibited in UUO + EDO mice (Table 2).

**Table 2:** Renal function parameters.

	sham	UUO	UUO + EDO
Plasma creatinine (mg/dL)	0.094 ± 0.002	0.163 ± 0.005**	0.136 ± 0.011**^,#^
Blood urea nitrogen (mg/dL)	24.15 ± 0.69	35.73 ± 1.46**	38.6 ± 2.59^**^

Values are expressed as means ± standard error of the mean (SEM). n = 7–16 per group. **p < 0.01 vs. sham; ^#^*p* < 0.05 vs. UUO.

### Effect of the FXa inhibitor on renal interstitial fibrosis and mRNA levels of extracellular matrix molecules

**Figure 2:**
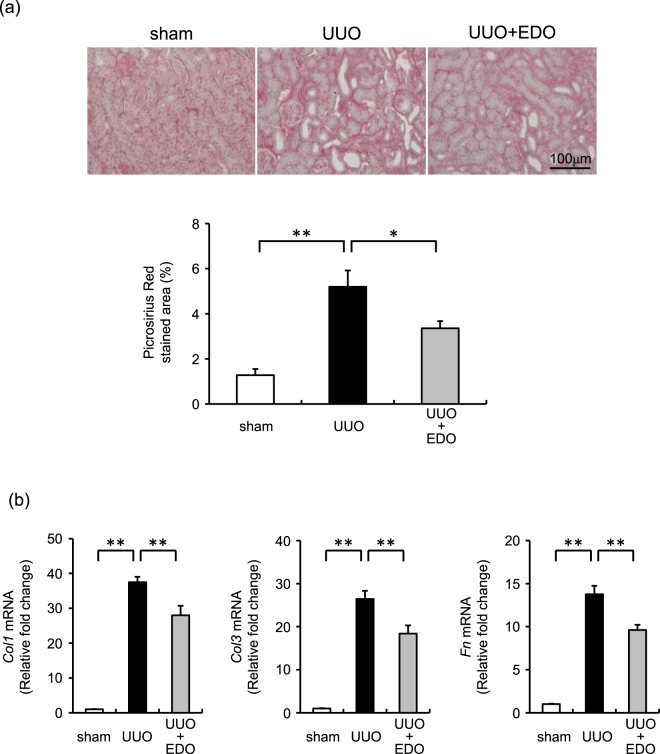
Effect of edoxaban on unilateral ureteral obstruction (UUO) -induced renal interstitial fibrosis. (**a**) Upper panel, representative histological findings in renal tissue stained with Picrosirius Red 7 days after operation; Lower panel, quantitative analysis of renal interstitial fibrosis at day 7. Sham operation with vehicle (sham; white bar), UUO with vehicle (UUO; black bar), and UUO with 50 mg/kg/day edoxaban (UUO + EDO; grey bar). Results are expressed as means ± standard error of the mean (SEM). *p < 0.05, **p < 0.01; n = 4–9 per group. (**b**) Changes in the mRNA levels of *Col1*, *Col3*, and *Fn* in the kidneys of mice at day 7. Results are expressed as means ± SEM. **p < 0.01. n = 8–13 per group.

We performed Picrosirius Red staining to examine renal interstitial fibrosis. After the operation, the progression of renal interstitial fibrosis was significantly greater in UUO mice than in sham mice, but this fibrotic progression was significantly mitigated in UUO + EDO mice (Fig. 2a). Consistent with changes in fibrosis, the renal expression levels of *Col1*, *Col3*, and *Fn* mRNA in UUO mice were also higher than those in sham mice, but these increases were significantly attenuated in UUO + EDO mice (Fig. 2b).

### Effect of the FXa inhibitor on renal interstitial macrophage infiltration and inflammatory cytokines

**Figure 3:**
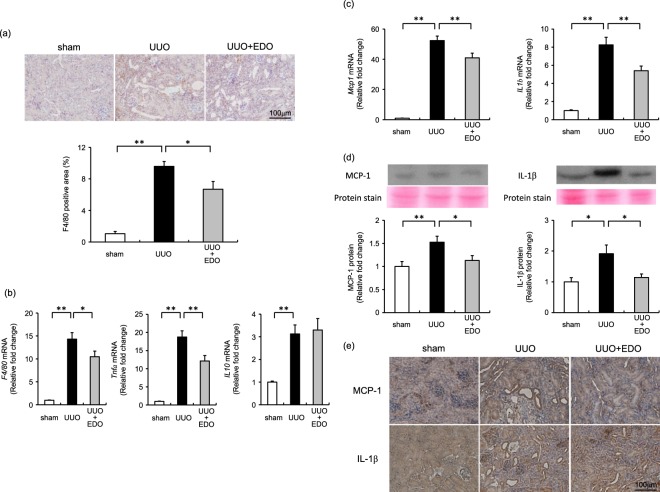
Renal interstitial macrophage infiltration and inflammatory cytokines. (**a**) Upper panel, representative immunohistochemical results for F4/80 antibodies 7 days after operation; Lower panel, quantitative analysis of the F4/80-positive area within the interstitial area at day 7. Sham operation with vehicle (sham; white bar), unilateral ureteral obstruction (UUO) with vehicle (UUO; black bar), and UUO with 50 mg/kg/day edoxaban (UUO + EDO; grey bar). Results are expressed as means ± standard error of the mean (SEM). *p < 0.05, **p < 0.01; n = 6–7 per group. (**b**) Changes in the mRNA levels of *F4/80*, tumour necrosis factor α (*Tnfα*), and interleukin-10 (*IL10*) in the kidneys of mice at day 7. Results are expressed as means ± SEM. *p < 0.05, **p < 0.01; n = 5–13 per group. (**c**) Changes in the mRNA levels of monocyte chemotactic protein (*Mcp1*), and interleukin-1β (*IL1β*) in the kidneys of mice at day 7. Results are expressed as means ± SEM. **p < 0.01. n = 6–13 per group. (**d**) Upper panel, representative images of MCP-1 and IL-1β protein staining in the kidney; Lower panel, semi-quantitative analysis of MCP-1 and IL-1β expression by densitometry (full-length blots are presented as Supplementary Figure 1). Results are expressed as means ± SEM. *p < 0.05, **p < 0.01. n = 7–15 per group. (**e**) Representative immunohistochemical results for MCP-1 and IL-1β antibodies 7 days after operation.

We studied macrophage infiltration by immunochemical staining and inflammatory cytokine expression by qRT-PCR to investigate the effects of an FXa inhibitor on inflammation in UUO-induced renal interstitial fibrosis. The increased area of interstitial macrophage infiltration in UUO mice was significantly greater than that in sham mice, but was significantly reduced in UUO + EDO mice (Fig. 3a). This result was consistent with the changes in *F4/80* mRNA levels, which were also significantly higher in UUO mice than in sham mice, but significantly reduced in UUO + EDO mice. The increase in TNF-α (an M1 marker) expression in UUO mice was reduced by edoxaban treatment. IL-10 (an M2 marker) expression was also increased in UUO mice, but was not affected by edoxaban treatment (Fig. 3b). Consistent with attenuated macrophage infiltration, UUO-induced upregulation of the expression of MCP-1 and IL-1β in UUO mice was also significantly reduced in UUO + EDO mice (Fig. 3c–e).

### Effect of the FXa inhibitor on the mRNA levels of transforming growth factor-β (TGF-β) and α-smooth muscle actin (αSMA)

**Figure 4:**
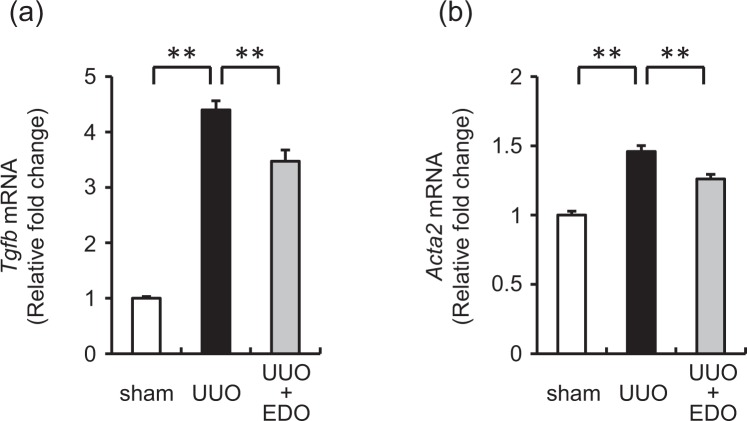
Effect of edoxaban on mRNA levels of transforming growth factor β (TGF-β) and α-smooth muscle actin (αSMA), a myofibroblast marker. Changes in the mRNA expression levels of *Tgfb* (**a**) and *Acta2* (**b**) in the kidneys of mice at day 7. Sham operation with vehicle (sham; white bar), unilateral ureteral obstruction (UUO) with vehicle (UUO; black bar), and UUO with 50 mg/kg/day edoxaban (UUO + EDO; grey bar). Results are expressed as means ± standard error of the mean (SEM). **p < 0.01. n = 8–13 per group.

TGF-β is a crucial factor in the development of tissue fibrosis. Therefore, we examined whether the protective effect of edoxaban on UUO-induced renal fibrosis was associated with TGF-β. The renal expression level of TGF-β was significantly greater in UUO mice than in sham mice, but this increase was significantly attenuated in UUO + EDO mice (Fig. 4a).

The expression of αSMA, a marker of activated fibroblasts (also known as myofibroblasts), was significantly higher in UUO mice than in sham mice, but this increase was significantly attenuated in UUO + EDO mice (Fig. 4b).

### Decrease in the occurrence of tubulointerstitial nephritis in response to FXa inhibitor treatment according to the FAERS database

Finally, to investigate the renoprotective effect of FXa inhibitors, including edoxaban, in real-world clinical practice, we used the FAERS database to estimate the occurrence of tubulointerstitial nephritis in patients treated with two types of anticoagulant drugs: FXa inhibitors and warfarin. The entire FAERS database contained 8,281,917 reports from January 2004 to September 2017, including 4,947 tubulointerstitial nephritis event reports. The reporting odds ratios (RORs) and 95% confidence intervals (CIs) of tubulointerstitial nephritis in patients treated with FXa inhibitors (edoxaban, rivaroxaban, or apixaban) or warfarin are summarized in Table 3. A significant inverse association with tubulointerstitial nephritis adverse events was found for FXa inhibitors (ROR, 0.65; 95% CI, 0.49–0.85), but not warfarin (ROR, 0.94; 95% CI, 0.76–1.16).

**Table 3:** Reporting odds ratio (ROR) for tubulointerstitial nephritis according to anticoagulant drug or drug class in the FAERS database.

	Cases	Non-cases	ROR	(95% CI)
Tubulointerstitial nephritis	4,947	8,276,970		
FXa inhibitors^a^	52	132,539	**0**.**65**	(**0**.**49–0**.**85**)
Warfarin	85	150,455	0.94	(0.76–1.16)

Bold text indicates statistically a significant ROR (upper limit of the 95% confidence interval <1). ^a^FXa inhibitors; one or more drugs from among edoxaban, rivaroxaban, and apixaban.

## Discussion

In this study, the FXa inhibitor edoxaban attenuated renal fibrosis as well as the mRNA levels of ECM components, such as collagen and fibronectin, in UUO mice. In addition, edoxaban also inhibited macrophage infiltration and inflammatory molecule expression. Furthermore, an analysis of the FAERS data revealed that FXa inhibitors are associated with a significant reduction in reports of tubulointerstitial nephritis in real-world clinical settings. These results suggest that FXa inhibitors suppress renal fibrosis via inhibition of inflammation by FXa inhibition.

FXa increases inflammatory molecule expression in many cell types, such as macrophages, endothelial cells, and podocytes^8,13,14^. Diabetes mellitus is associated with increased renal *FX* mRNA and urinary FXa activity^13^. In asthmatic mice, *FX* mRNA is elevated in the lungs and bronchoalveolar lavage fluid macrophages, and FXa activity is increased in the bronchoalveolar lavage fluid^7^. *Par1* and *Par2* mRNA levels are higher in arteriosclerosis model mice than in wild-type mice^8^. These findings are consistent with our observation of increased *FX*, *Par1* and *Par2* mRNA levels in UUO mice. UUO mice exhibit renal fibrosis in one kidney and maintain normal function in the other. In this study, the activity of plasma FXa did not differ between sham mice and UUO mice, but the expression levels of FX and TF were elevated in the UUO kidney. Therefore, the confined increase in renal blood coagulation factors involves inflammation and renal fibrosis in the UUO kidney. Edoxaban administration significantly suppressed the increased levels of plasma creatinine in UUO mice, suggesting that edoxaban has a renoprotective effect.

Tubulointerstitial fibrosis is a hallmark of CKD. FXa stimulates fibroblast procollagen production and proliferation via PAR-1 activation^20^. Moreover, PAR-1 antagonism protects against fibrosis in a rodent model of liver injury^21^. In IgA nephropathy renal biopsies, increased expression of PAR-2 has been detected, and *Par2* mRNA levels are directly correlated with the extent of interstitial fibrosis^6^. Renal tubulointerstitial fibrosis was attenuated in mice lacking PAR-2 (*F2rl*1^−*/*−^)^22^. Furthermore, FXa stimulates profibrotic responses in fibroblasts via PAR-2 activation^23^. In this study, we demonstrated that edoxaban suppresses renal interstitial fibrosis as well as *Col1*, *Col3* and *Fn* mRNA expression. These results suggest that edoxaban attenuates renal interstitial fibrosis by inhibiting the FX–PAR pathway.

FXa also stimulates proinflammatory responses in fibroblasts via PAR-2 activation^23^. Macrophage infiltration and inflammatory molecule expression are observed in UUO mice^24^. We demonstrated that edoxaban suppresses macrophage infiltration into the kidneys of UUO mice. Although the M1 marker TNF-α was suppressed in UUO + EDO mice compared with UUO mice, no difference was observed between UUO mice and UUO + EDO mice in the M2 marker IL-10. Edoxaban also suppressed inflammatory molecule (MCP-1 and IL-1β) expression. Edoxaban may have its anti-inflammatory effects.

TGF-β is a cytokine that promotes fibrosis. In the present study, edoxaban suppressed *Tgfb* mRNA in UUO + EDO mice. Activation of PAR-2 by FXa increases TGF-β expression in both mesangial and tubular cells^6^. Thus, the inhibition of TGF-β signalling by FXa inhibitors may partially contribute to the inhibition of renal interstitial fibrosis. In addition, myofibroblasts are a major source of ECM production^25^, and fibroblasts can be activated by various cytokines including TGF-β^26^. In the present study, edoxaban suppressed *Acta2* mRNA in UUO + EDO mice. These results suggest that edoxaban inhibits renal fibrosis by inhibiting the activation of TGF-β and the transition of fibroblasts to myofibroblasts.

Recent clinical research has shown that the blood levels of the blood coagulation factor FVIII is significantly higher in patients with CKD than in healthy controls, and that these levels increase with CKD progression^4^. Therefore, we used the FAERS to test the hypothesis that FXa inhibitors have renoprotective effects. Several studies have successfully discovered novel therapeutic strategies for antipsychotic-induced hyperglycaemia and depression-like behaviour based on analyses of FAERS data^27,28^. As predicted, the FAERS data analysis demonstrated that the patients treated with FXa inhibitors exhibited significantly lower rates of tubulointerstitial nephritis than those of patients not treated with FXa inhibitors. This result suggests that FXa inhibitors, at clinically relevant doses, could help prevent the onset of tubulointerstitial nephritis in humans. Interestingly, there was no difference in the incidence of tubulointerstitial nephritis between warfarin-treated patients and patients who did not receive warfarin. These results suggest that the inhibitory effect against tubulointerstitial nephritis is specific to FXa inhibitors. Our FAERS analysis had some limitation, e.g. the reported drug side effects. Despite the limitations inherent to spontaneous reporting systems, the results of this FAERS database analyses support the finding that edoxaban prevents renal fibrosis in an experimental mouse model of tubulointerstitial fibrosis and indicate that FXa inhibitors could exert a renoprotective effect in clinical practice. Several FXa inhibitors are used in clinical settings and show renal protective effects. Similar to the results of our study, edoxaban and fondaparinux have protective effects against diabetic nephropathy^13,29^. In addition, rivaroxaban has a protective effect against atherosclerotic lesions in apolipoprotein E-deficient mice, and neointima formation after wire-mediated vascular injury, although they do not exhibit renoprotective effects^8,14^. Taken together, the effects of FXa inhibitors might be related to drug class, rather than to specific drugs. However, it has recently been reported that the risk of adverse renal outcomes is lower for direct oral anticoagulants than warfarin in patients with atrial fibrillation. In that study, rivaroxaban inhibited the renal function decline, but apixaban had no affect^30^. Therefore, the renal protective effect of FXa inhibitors may differ among drugs. Further investigations are necessary to clarify these differences.

In conclusion, the results of this study suggest that the FXa inhibitor edoxaban attenuates renal fibrosis by regulating inflammatory responses, indicating that it might exert a protective effect against CKD. FXa could be an effective therapeutic target for the management of pathological conditions associated with renal fibrosis. However, studies using the CKD model are necessary in the future.

## Methods

### Chemicals

Edoxaban was provided by Daiichi Sankyo Co., Ltd. (Tokyo, Japan). The following commercially available antibodies were used in this study: anti- PAR-1 antibody (Santa Cruz Biotechnology, Santa Cruz, CA, USA); anti- PAR-2 antibody (Santa Cruz Biotechnology); anti-rat F4/80 antibody (AbD Serotec, Oxford, UK); anti- MCP-1 antibodies (Cell Signaling Technology, Danvers, MA, USA); anti- IL-1β antibodies (Santa Cruz Biotechnology).

### Animal experimentation and treatment

The guidelines of the Animal Research Committee of the University of Tokushima Graduate School were followed, and the study protocol was approved by the Tokushima University Institutional Review Board for Animal Protection (Permit Number: 13095).

Male C57BL/6J mice of 7 to 9 weeks old were obtained from CLEA Japan Inc. (Tokyo, Japan). They were given free access to water and food (Type NMF; Oriental Yeast, Tokyo, Japan) during the study. UUO was performed by ligation of the left ureter with 3–0 silk at two proximal site points under pentobarbital anaesthesia as previously described^24^. In sham mice, the left ureter was exposed but not ligated. Mice were divided into three groups: sham, UUO, and UUO + EDO (50 mg/kg/day edoxaban via oral gavage for 7 days immediately after operation). The edoxaban dose was determined as previously described^13^.

### Real-time PCR

Extraction of total RNA, cDNA synthesis, and quantitative RT-PCR were performed as previously described^31^. In brief, total RNA was extracted with ISOGEN (NIPPON GENE Co., Ltd., Tokyo, Japan), and cDNA was synthesized using the PrimeScript RT Reagent Kit with gDNA Eraser (Takara Bio, Inc., Otsu, Japan) according to the manufacturer’s instructions. Quantitative RT-PCR was performed using the CFX Connect Real-Time PCR Detection System (Bio-Rad Laboratories, Hercules, CA, USA) with THUNDERBIRD SYBR qPCR Mix (TOYOBO CO., LTD., Osaka, Japan). The primer sets are noted in Table 4.

**Table 4:** Sequence of the primers used for real-time PCR.

Target gene	Forward (5′-3′)	Reverse (5′-3′)
*mFX*	GGCAGGCTCTGCTCATTAAC	TTCCGATCACCTACCCTCAC
*mTF*	ACAATTTTGGAGTGGCAACC	TCACGATCTCGTCTGTGAGG
*mPar* *1*	GTTGATCGTTTCCACGGTCT	GCAGACGATGAAGATGCAGA
*mPar*2	AACATCACCACCTGTCACGA	GCACGTAGGCAGATGCAGTA
*mCol1*	GAGCGGAGAGTACTGGATCG	GTTCGGGCTGATGTACCAGT
*mCol3*	ACCAAAAGGTGATGCTGGAC	GACCTCGTGCTCCAGTTAGC
*mFn*	ACAGAGCTCAACCTCCCTGA	TGTGCTCTCCTGGTTCTCCT
*mF4/80*	CTGTAACCGGATGGCAAACT	CTGTACCCACATGGCTGATG
*mTnfa*	ACGGCATGGATCTCAAAGAC	GTGGGTGAGGAGCACGTAGT
*mIL* *10*	GCTCTTACTGACTGGC	CGCAGCTCTAGGAGCA
*mMcp1*	GGAGCTCATGATGTGAGCAA	GACCAGGCAAGGGAATTACA
*mIL1b*	CAGGCAGGCAGTATCACTCA	TGTCCTCATCCTGGAAGGTC
*mTgfb*	TGAGTGGCTGTCTTTTGACG	AGCCCTGTATTCCGTCTCCT
*mActa2*	ACATCTGCTGGAAGGTGGAC	GCATGCAGAAGGAGATCACA
*36B4*	GCTCCAAGCAGATGCAGCA	CCGGATGTGAGGCAGCAG

### FXa activity

Plasma FXa levels were measured using the Mouse Coagulation FXa ELISA Kit (MyBioSource, Inc., San Diego, CA, USA). The assay was performed according to the manufacturer’s instructions.

### Renal function parameters

Plasma creatinine (enzyme method) and BUN (urease-GLDH method) levels were measured by Oriental Yeast Co., LTD. (Shiga, Japan).

### Identification of collagen

Picrosirius Red staining was used to evaluate fibrosis. Procedures were performed according to the manufacturer’s instructions. Briefly, the excised kidney was fixed with 4% paraformaldehyde and embedded in paraffin. A 5-μm-thick sample was stained with Picrosirius Red and evaluated. Fourteen fields were randomly selected in the renal cortex area. Fibrotic areas were quantified using ImageJ 1.38x (National Institutes of Health, Bethesda, MD, USA).

### Immunohistochemistry

Immunohistochemical detection was performed as described previously^24^. Paraffin-embedded kidney samples were cut into 5-μm sections and mounted on slides. After deparaffinization, sections were incubated for 10 min in 0.01 M citrate buffer at 95 °C for antigen retrieval. The sections were then incubated overnight with the primary antibody at 4 °C. Antibody distribution was visualized using a streptavidin-biotin complex assay and a DAB substrate kit (LSAB+ Kit Universal; Dako Japan, Tokyo, Japan). To evaluate renal macrophage-infiltration, 14 fields were randomly selected in the renal cortex area. The macrophage positive area was expressed as a percentage of the whole area, excluding the tubular lumen, glomeruli, and vessels, using ImageJ 1.38x.

### Western blot analysis

Western blotting was used to confirm protein expression. Briefly, extracts from kidneys were homogenized in T-PER (Thermo Fisher Scientific, Inc., Waltham, MA, USA) with a protease inhibitor cocktail (Roche Applied Science, Indianapolis, IN, USA) and a phosphatase inhibitor (Roche Applied Science) as previously described^24^. The immunoblot bands were subjected to a semi-quantitative analysis by densitometry using ImageJ 1.38x.

### FAERS data source

FAERS includes demographic and administrative, drug, reaction, and patient outcome information. The adverse event terms correspond to the Medical Dictionary for Regulatory Activities (MedDRA) v18.1. The drug names were converted to the generic name from the brand names using the DrugBank database (www.drugbank.ca) as a reference source for the batch conversion. Duplicate reports were excluded in accordance with FDA recommendations. MySQL software (version 5.7.21) was used to build a database that integrated the FAERS database.

### Statistical analysis

Values are expressed as means ± standard error of the mean (SEM). The significance of each difference was evaluated using the *t*-test for comparisons between two groups or using post-hoc Tukey-Kramer tests for comparisons among three groups. Values of *p* < 0.05 were accepted as statistically significant.

ROR was developed to detect drug-associated adverse events in the spontaneous reporting database^32–34^. To calculate the ROR, all reported adverse events associated with nephritis were defined as “cases” and all other reported adverse events as “non-cases”. The RORs were calculated from two-by-two contingency tables of counts that indicated the presence or absence of a particular anticoagulant drug or drug class and the tubulointerstitial nephritis adverse event in the case reports. These are expressed as point estimates with 95% CIs. The inverse signal was defined as the upper limit of the 95% CI for an ROR of <1^35^.

### Data availability

All data generated or analysed during this study are included in this published article.

## Electronic supplementary material

Supplementary Figure 1
