# Supplementary material for: Renoprotective effects of a factor Xa inhibitor: fusion of basic research and a database analysis
Source: Sci Rep. 2018 Jul 18;8:10858. doi: 10.1038/s41598-018-29008-2 (PMC6052035; doi:10.1038/s41598-018-29008-2)

Renoprotective effects of a factor Xa inhibitor: fusion of basic research and a database analysis

Yuya Horinouchi[1*,¶], Yasumasa Ikeda[1*,¶], Keijo Fukushima[2], Masaki Imanishi[3], Hirofumi Hamano[1,3], Yuki Izawa-Ishizawa[1],Yoshito Zamami[3,4], Kenshi Takechi[5], Licht Miyamoto[6], Hiromichi Fujino[2], Keisuke Ishizawa[3,4], Koichiro Tsuchiya[6], and Toshiaki Tamaki[1]

[1]Department of Pharmacology, [2]Department of Pharmacology for Life Sciences, Institute of Biomedical Sciences, Tokushima University Graduate School, Tokushima, Japan

[3]Department of Pharmacy, Tokushima University Hospital, Tokushima, Japan

[4]Department of Clinical Pharmacology and Therapeutics, Institute of Biomedical Sciences, Tokushima University Graduate School, Tokushima, Japan

[5]Clinical Trial Center for Developmental Therapeutics, Tokushima University Hospital, Tokushima, Japan

[6]Department of Medical Pharmacology, Institute of Biomedical Sciences, Tokushima University Graduate School, Tokushima, Japan

¶ These authors contributed equally to this work.

* Corresponding authors

Supplementary Figure 1: Full-length blots for use as representative figures in the manuscript.

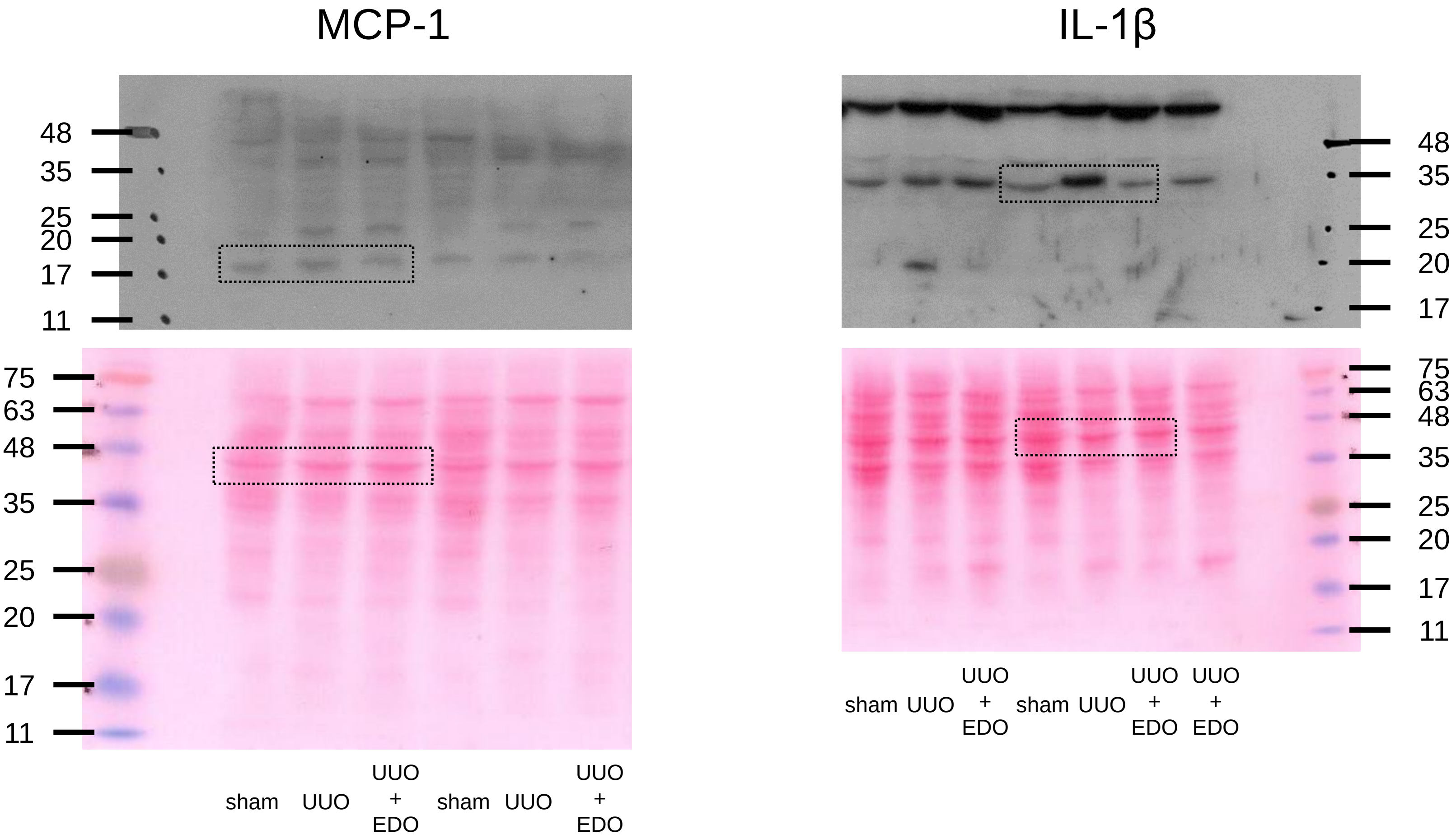

Supplement: Supplementary file 1 — Supplementary Figure 1 [file 41598_2018_29008_MOESM1_ESM.pdf]
